# Operational status of the X-ray powder diffraction beam­line at the SESAME synchrotron

**DOI:** 10.1107/S1600577521012820

**Published:** 2022-01-17

**Authors:** Mahmoud Abdellatief, Mohammad Al Najdawi, Yazeed Momani, Basil Aljamal, Anas Abbadi, Messaoud Harfouche, Giorgio Paolucci

**Affiliations:** a SESAME Synchrotron, King Hussein Bin Talal St/Box 7, Allan 19252, Jordan; b Elettra-Sincrotrone Trieste SCpA, Strada Statale 14 – km 163.5 in AREA Science Park, Basovizza, Trieste 34149, Italy

**Keywords:** SESAME synchrotron, X-ray diffraction, materials science, beam­line, line profile analysis, crystal structure

## Abstract

The Materials Science (MS) beam­line at SESAME (Synchrotron-light for Experimental Science and Applications in the Middle East), dedicated to the X-ray powder diffraction technique, started its operational phase in December 2020 by hosting its first users. The MS endstation comprises a two-circle diffractometer coupled with a PILATUS 300K area detector, with which direct beam images are collected and compared with the initial ray-tracing simulation results. We present a detailed description of the beam­line components and the experimental characterization of the main instrumental parameters relying on the instrumental profile and the angular resolution. A representative example for microstructure investigations of a nanocrystalline material is demonstrated.

## Abstract 2

The Materials Science (MS) beam­line at SESAME, dedicated to the X-ray powder diffraction technique, started its operational phase in December 2020. A detailed description of the beam­line components and the experimental characterization of the main instrumental parameters are presented.

## 1. Introduction

X-ray diffraction (XRD) beam­lines are commonly among the high-priority beam­lines because of their wide range of applications in diverse fields, such as materials science, biology, pharmacology and cultural heritage. The X-ray powder diffraction (XRPD) technique may be applied for materials phase identification, qu­anti­tative phase analysis and microstructural analysis (*e.g.* structural defects and crystal size analysis), and for kinetic studies. The MS beam­line is the third operational beam­line at SESAME, following on from the XAFS/XRF beam­line, which started its operational phase in November 2017, and the IR beam­line, which began operation in 2018.

The MS beam­line utilizes optical components previously installed at the Swiss Light Source (SLS) (Gozzo *et al.*, 2004 ▸; Patterson *et al.*, 2005 ▸). However, some modifications have been introduced to the beam­line design in order to match the characteristics of the SESAME storage ring (Abdellatief *et al.*, 2017 ▸). The MS beam­line is based on a wiggler source producing a high flux of the order of 10^13^ photons s^−1^ at the sample location at 10 keV (calculated for a full current of 400 mA). In addition, the beam­line energy range is 5–25 keV, the energy resolution is about 2 eV and the effective beam size at the sample is 300 µm × 2800 µm. The beam­line endstation characteristics and specifications are important for finding the best experimental conditions for each experiment, and also for figuring out the best capabilities for the beam­line. The best parameters of the beam­line have already been defined and analyzed theoretically through a ray-tracing study (Abdellatief *et al.*, 2017 ▸) using the *SHADOW3* code (last distribution *Sha­dow­Oui*; Rebuffi & Sánchez del Río, 2016 ▸). The main parameters resulting from the ray-tracing study are summarized in Table 1 ▸. Herein, a revised layout is presented for the beam­line, a full experimental analysis is carried out for the beam­line endstation parameters and a comparison is made with the theoretical ray-tracing results. In addition, some preliminary experiments measured at the beam­line are outlined.

## 2. Outline for MS layout

The MS beam­line consists of three main sections: the front-end, the optics hutch and the experimental station, located after the wiggler source which consists of an array of NdFe:B permanent magnets (Gozzo *et al.*, 2004 ▸; Patterson *et al.*, 2005 ▸). The main components of the beam­line and their corresponding distances from the wiggler centre are tabulated in Table 2 ▸.

### 2.1. Front-end section

The beam­line front-end is the first main section and is located directly after the wiggler source inside the SESAME storage ring along the I09 straight section. The minimum magnetic gap is 12 mm, which was chosen based on the minimum vacuum chamber height (10 mm) during the first operation period, and the maximum magnetic field of the wiggler at 12 mm is 1.38 T. The magnetic gap of the wiggler was originally 8 mm at the SLS, which allowed the SLS-MS beam­line to reach 40 keV, but this gap was technically difficult to reach at SESAME.

**Figure 1:**
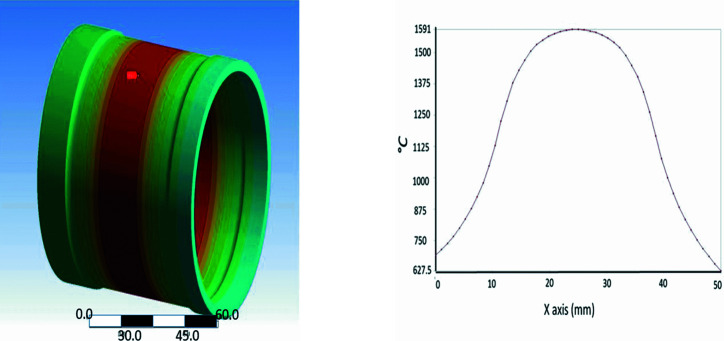
Temperature distribution on the rotating filter by finite-element analysis.

The front-end has several functions, such as defining the beam angular acceptance range (1.5 mrad × 0.23 mrad), which is achieved using a fixed mask component. Another necessary function of the front-end is to deal with the high photon power produced by the wiggler source (about 6000 W). This is accomplished by a rotating glassy graphite filter with a 1 mm wall thickness (*i.e.* 2 mm filter thickness in total), which is rotating continuously in order to distribute the radiation power over a larger area. Dealing with the high photon power, normally found in the low X-ray energy range, is necessary to protect the beam­line optics. Therefore, filter rotation and cooling are linked to the beam­line Equipment Protection System (EPS). The fixed mask reasonably cuts a large amount of power, about 3850 W, with about 2150 W being transmitted through and received at the rotating filter. Cooling of the filter is performed through nondirect contact by a radiation mechanism, achieved by cooling a copper enclosure surrounding the filter. For ensuring the practicality of this design, a finite-element analysis (FEA) was applied to obtain the temperature distribution on the graphite rotating filter (Fig. 1 ▸) and also on the copper enclosure. The FEA analysis predicts that the filter absorbs 1.54 kW at 400 mA, which raises its temperature to 1591°C and raises the temperature of the copper enclosure to 135°C. These values agree with similar studies on the filter when it was in operation at the SLS (Heidenreich & Patterson, 2007 ▸).

A water-cooled photon shutter, utilized to control the beam­line operation, is located between the fixed mask and the rotating filter. The white beam slits are located after the rotating filter to reduce the beam scattering that might be produced after the rotating filter and the fixed mask. Lastly, a tungsten (W) 200 mm-long radiation stopper, in cylindrical form, is used to block the high-energy radiation (*e.g.* gas bremsstrahlung radiation).

### 2.2. Optical section

The MS beam­line optical layout consists of a cylindrically collimated rhodium-coated mirror aligned and fixed to a 3 mrad grazing angle reflecting the beam upwards. A Kozhu double-crystal fixed-exit monochromator (DCM) is located 2 m from the collimating mirror centre. The DCM is equipped with a pair of Si(111) crystals, where the second crystal is sagittally bendable to focus the beam horizontally at the sample location. Then a second cylindrical rhodium-coated mirror similar to the collimating mirror (face down) is placed so as to bring the beam back to its original horizontal trajectory and also to focus the beam vertically at the location of the sample.

The optical components are aligned and tuned to select the beam energy and to optimize the beam properties at the location of the sample, in terms of beam size, flux and shape.

The first mirror was aligned during the beam­line commissioning and fixed at 3 mrad. This reflects the beam up with an angle twice that of the grazing angle relative to the horizontal plane. The beam then hits the first Si(111) crystal of the monochromator, for which the angle is tuned to select the desired beam energy. A second crystal is then placed with certain and precisely calculated vertical and horizontal displacements from the first crystal to deliver a fixed-exit beam. A full in-house hardware/software control system was developed for the monochromator at SESAME for controlling the monochromator motion system for both motion and EPS controls. The second crystal brings the beam back to the semihorizontal trajectory (same trajectory of the beam out from the first mirror ‘6 mrad’), and also focuses the beam horizontally at the location of the sample. It is possible to alter the tuning pitch, roll and yaw angles of the second crystal to optimize the beam flux, position and shape at the endstation.

Having some diagnostic devices between the optical com­ponents is necessary and was very useful during the commissioning phase of the beam­line. These devices are mainly a tungsten wire monitor between the first mirror and the monochromator with an encoded motor to define the direct vertical beam position and shape, as well as the shift in distance of the beam due to the first mirror reflection. The second important diagnostic device for the optics is a homemade phospho­rus screen placed in a pneumatic holder between the monochromator and the second mirror. Then, different from the original optics layout of the SLS-MS beam­line, slits were added to control and limit any undesired scattering of the beam. Three optical modes of operation were already in use at the SLS–MS beam­line, but only the normal monochromatic beam is in use at the SESAME MS beam­line.

With the MS beam­line source and optics, the photon energy of the beam­line can be varied from a low of about 5 keV up to about 25 keV. However, due to the low reflectivity of the Rh mirror at high energy, it is recommended to stay below the Rh *K*-edge of 23.22 keV. However, an energy range between 12 and 21 keV is considered a reasonable compromise of flux and energy.

### 2.3. Experimental section

**Figure 2:**
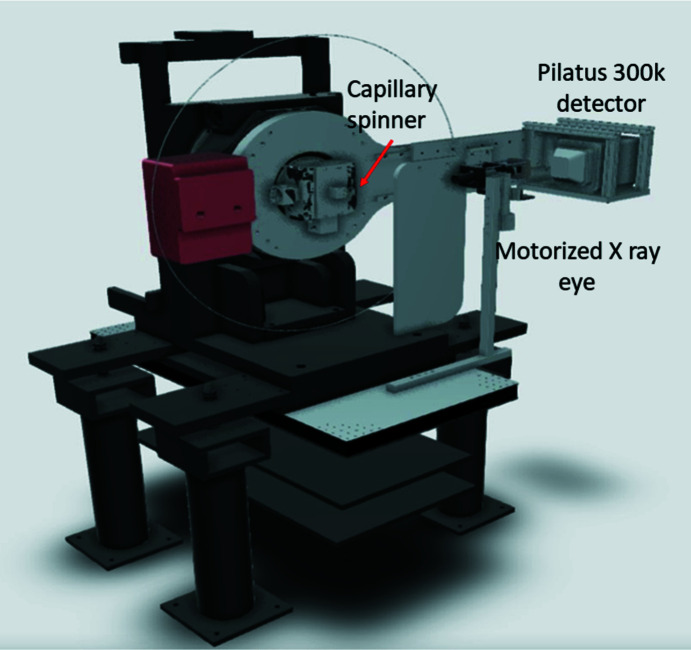
The MS beam­line two-circle diffractometer.

The MS experimental station consists of a refurbished two-circle diffractometer (manufactured by the Crystal Logic company; see Fig. 2 ▸) previously operated at the I19 beam­line of the Diamond synchrotron for single-crystal diffraction experiments. Before refurbishment, it contained four rotation movements, three for the sample, namely theta, chi and kappa, and one for the detector named 2theta (Huber 440 rotary stage).

Numerous modifications were made to the diffractometer to meet the requirements of powder diffraction applications. The first modification was to elongate the 2theta arm to increase the sample-to-detector distance. The active surface of the new detector is then located at about 735 mm from the diffractometer centre of rotation (position of the sample). The original manual XY translational stage attached to the theta sample air-bearing rotation stage was then replaced by a motorized translational XY stage (Huber 5102.20 stage). This original manual XY stage was attached on the theta rotary to hold another kappa stage necessary for the single crystal; this was removed and replaced by a homemade spinner that was attached to the motorized XY translational stage.

Knowing the exact position of the detector is crucial feed-back information to record, and hence an absolute rotary encoder was installed on the 2theta stage. The basic rotary stages for the sample and the detector provide the necessary flexibility to perform most XRPD experiments.

Glass capillaries mounted on a standard goniometer head fixed on the spinner are used for the XRD measurements in transmission mode. Working at high and low temperatures is possible by heating (to ∼1300 K) and cooling (to ∼95 K) using a hot-gas blower and a liquid-nitro­gen cryostat, respectively.

A PILATUS 300K detector donated by the DECTRIS company to SESAME is the main detection system at MS beam­line. It gives a very good compromise between experimental time and angular resolution if it is used in the proper geometrical setup by increasing the sample–detector distance.

A set of pneumatic aluminium filters followed by a microslit system are placed before the diffractometer and are used to control the incoming beam flux and shape. A small ionization chamber is available after the slits in order to optimize the beam intensity and to keep track of the flux changes.

A homemade X-ray eye is fixed at the end of the experimental hutch. It is composed of an X-ray phospho­ric screen (Codec) followed by a lead transparent glass window to protect the camera.

The control of the diffractometer was developed by SESAME’s control group and manages the motion control based on an EPICS system and the experimental data acquisition. The output obtained by the PILATUS detector is a TIFF file for each frame, with one frame covering about 6.4°.

**Figure 3:**
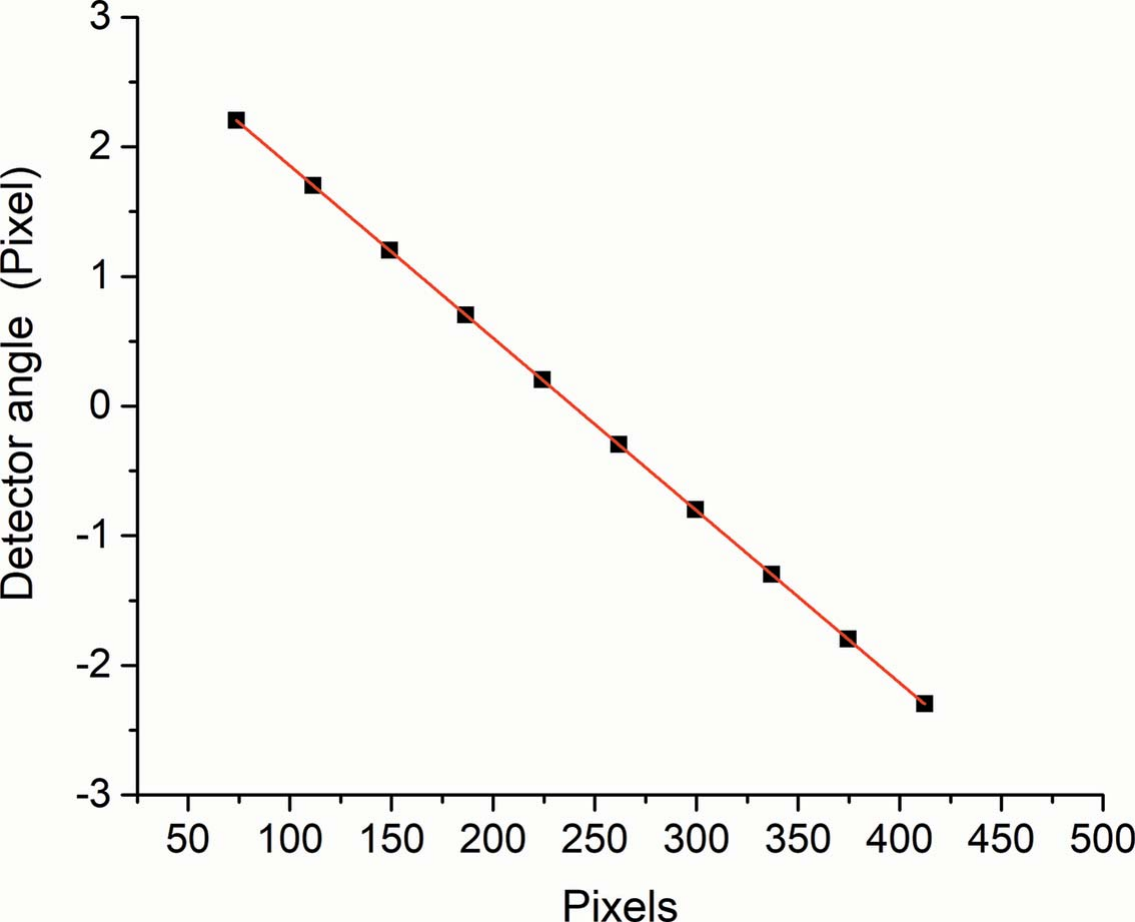
The relationship between the detector angles and the related pixels.

The process to extract the diffraction data from each frame image can be done considering the geometrical corrections (*e.g.* detector tilt angles). Many types of software can be used for such a calibration method, but having a small area detector at a fixed distance from the sample limits the number of diffracted peaks that can be collected in one image; hence, applying such a calibration method is quite complicated with a low number of peaks and can lead to peak shifts. Another method for data extraction was checked by tracking a certain silicon standard peak along the detector pixels (integration values X = 1–487 and Y = 305) in order to obtain an experimental relationship between pixels and the angle on the detector relying on the 2theta encoder reading. The best fit using equation (1) can then be applied to any frame, as shown in Fig. 3 ▸, from which the absolute diffraction angle on the detector can be obtained by adding the 2theta arm angle according to Equation (2). This data extraction method was applied at different peaks over the full range of 2theta and it was satisfactory enough.

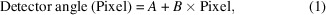




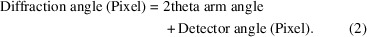




Several types of software can be used to process the TIFF images using the previous method [*e.g. DAWN* software (Filik *et al.*, 2017 ▸) and *ImageJ* software], if the proper list of steps is followed. Therefore, in order to save time and effort, a macro script through *ImageJ* software was written to process any number of TIFF images saved in a certain directory and create an XY data file of absolute diffraction angles and corresponding intensities. In the macro script, it is possible to limit the extracted detector angles from each image by defining the initial and final pixels active for integration. Hence, only 5° from each image frame can be extracted directly by the macro process. A simple executable Python-based code is then used to merge all data files for each experiment to create one merged file (Zubi & Abdellatief, 2021 ▸).

## 3. Beam parameters at the endstation

### 3.1. Beam shape

In this section, a comparison is made between the beam shape and size at the location of the sample resulting from the ray-tracing study and the real experimental beam.

*ShadowOui* (Rebuffi & Sanchez del Rio, 2016) was used to study the beam properties at the sample location and to optimize the beam­line optics theoretically (Abdellatief *et al.*, 2017 ▸). From the ray-tracing analysis, and in order to mimic the experimental setup of the beam­line, many physical parameters of the beam­line were taken into account, not only the geometrical factors of the beam­line optical components but also the absorption coefficient of the screens/filters, the reflectivity of the mirrors/multilayers, the diffraction profile of the crystals and the slope errors of the mirrors. The beam size resulting from the ray tracing is about 2.8 mm × 0.3 mm, referring to the beam full width at half-maximum (FWHM) at 15 keV.

**Figure 4:**
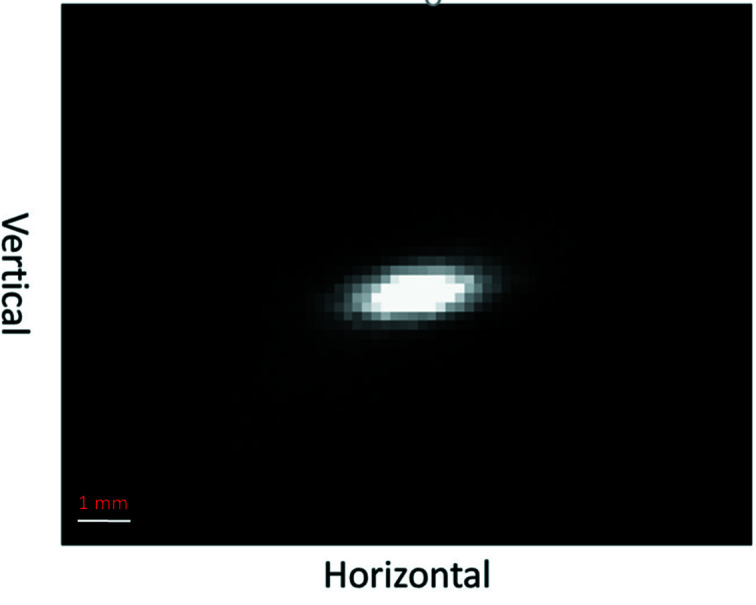
The direct focused photon beam collected on a PILATUS 300K detector at 15 keV.

A direct beam was measured on the PILATUS detector using prefilters to decrease the direct beam flux. The direct focused beam is shown in Fig. 4 ▸.

**Figure 5:**
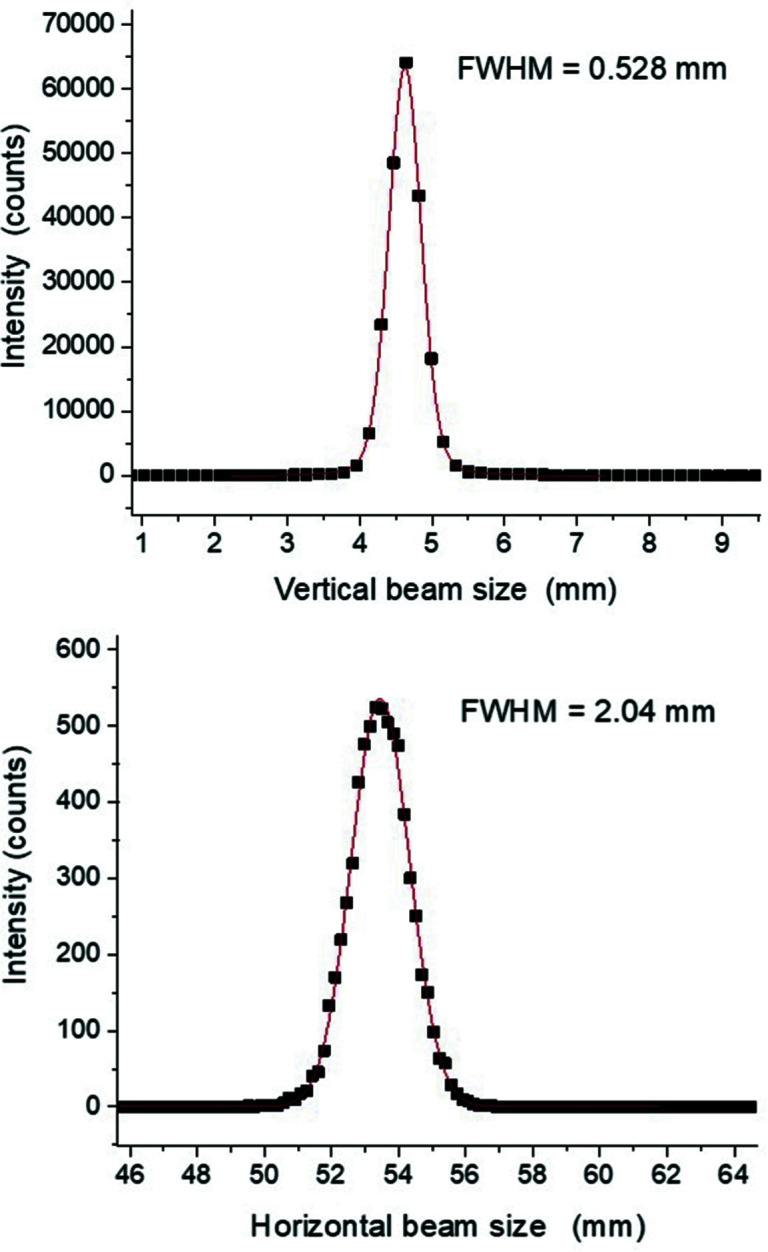
Vertical and horizontal focused beam profiles at the detector location.

An integration over the beam image was applied to obtain the exact size and profile of the beam; the effective FWHM for the horizontal beam was measured to be 2.04 mm (Fig. 5 ▸). This size is very close to that simulated from the ray tracing; on the other hand, the effective vertical size is 0.5 mm, which is slightly larger than the simulation result. This could be the result of the direct unfocused beam from the source that was found to have a strong tilt which cannot be corrected by the beam­line optics.

**Figure 6:**
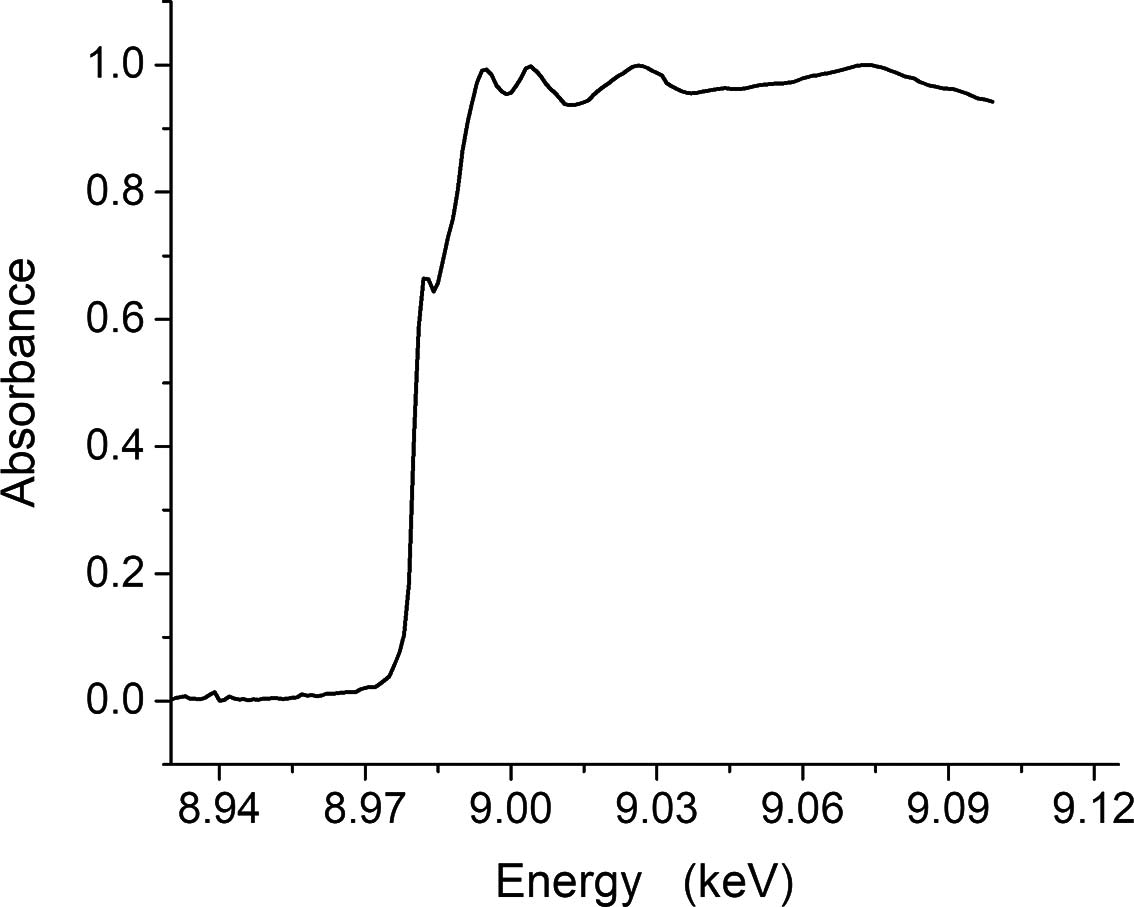
X-ray absorption fine-structure spectrum at the Cu edge.

**Figure 7:**
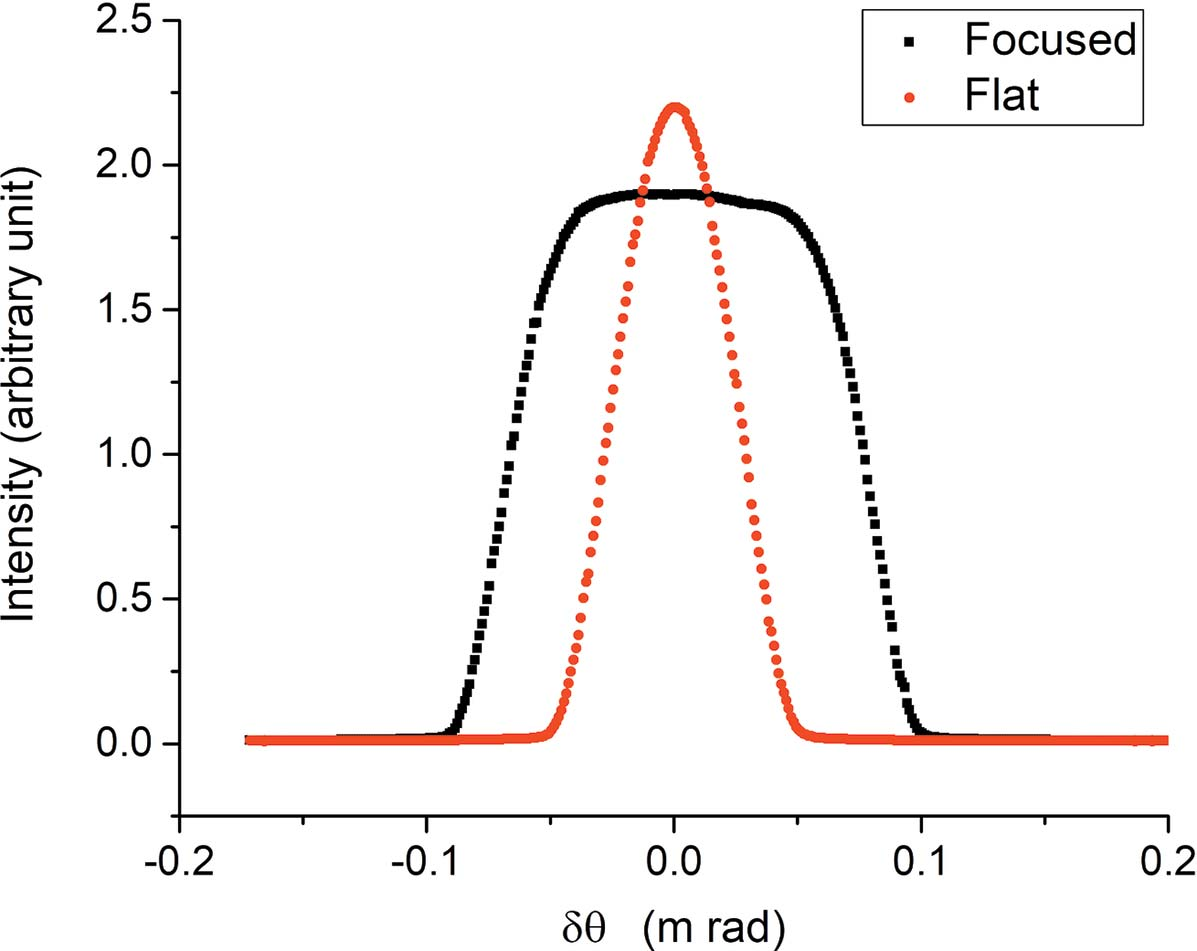
The rocking curve at 12 keV for the flat and focused crystals.

A theoretical calculation for an Si(111) crystal intrinsic energy resolution is Δ*E*/*E* ≅ 2 × 10^−4^. A scan of the energy for a Cu foil standard around the Cu *K*-edge (8979 eV) was recorded, after focusing the beam­line optics, to collect an X-ray absorption near-edge structure (XANES) spectrum, as shown in Fig. 6 ▸. Special attention was given to the energy step size during the scan, particularly at the pre-edge and the white line in order not to lose the shape of the features of these bumps. All the XANES features, including the pre-edge and the white line, are clearly visible and show a well-resolved spectrum. A rocking curve spectrum was measured at 12 keV (Fig. 7 ▸) for a focused crystal, while the experimental measured broadening was 27 arcsec FWHM, which supports the XANES results and demonstrates the good energy resolution obtained by the monochromator.

From the ray-tracing results, the simulated energy distribution emerging from the double-crystal monochromator at 12 keV has a bandwidth of 1.7 eV, but the tails of the distribution comprehend a ∼10 eV range (Abdellatief *et al.*, 2017 ▸). The experimentally obtained bandwidth of the sagittal Si crystal at 12 keV was 9 eV which is very similar to the ray-tracing result.

Another rocking curve was measured at 12 keV for the flat Si crystal (Fig. 7 ▸), showing a very symmetric and narrow bandwidth of 3.5 eV FWHM. The current rocking curve results agree with a similar investigation in the literature (Mikula & Vrána, 2015 ▸), in which the rocking curve behaviour for an Si bendable crystal was measured at different focusing distances. The increase of the rocking curve FWHM resulted from an increased curvature of the crystal and is attributed to an increase of the effective mosaicity of the second crystal.

### 3.2. Instrumental angular resolution

**Figure 8:**
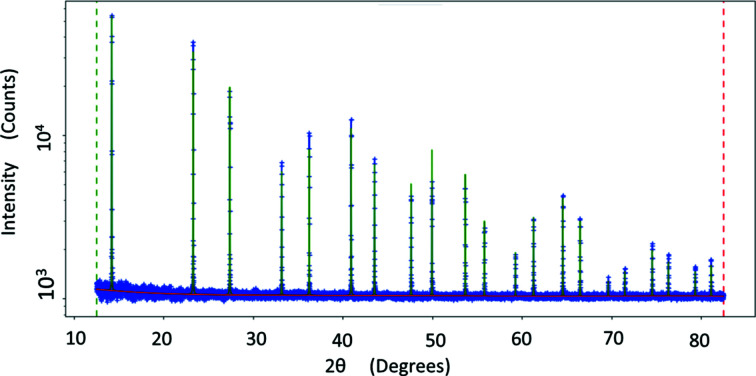
Silicon standard (NIST 640f) diffraction pattern at 16 keV.

**Figure 9:**
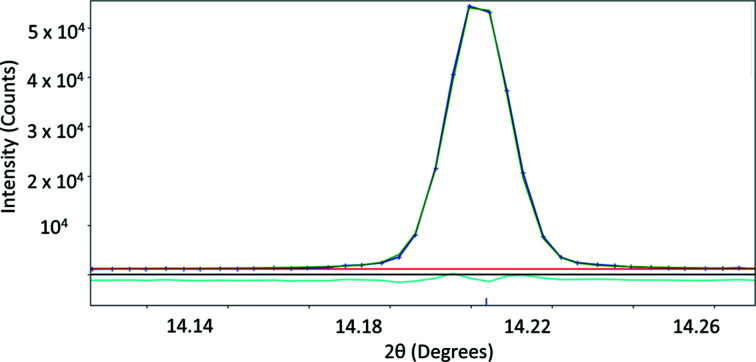
Silicon diffraction profile shape and fitting.

Several diffraction experiments for a silicon standard NIST SRM 640f, filled in a 0.5 mm borosilicate capillary, were measured and analyzed at different energies to determine the instrumental profile function of the MS beam­line. After processing the images created by the PILATUS 300K detector at each detector angular position, the diffraction peaks were then fitted using the pseudo-Voigt function to obtain the best FWHM values. Caglioti functions (Caglioti *et al.*, 1958 ▸) for FWHM predict that the instrumental FWHM obeys a second-order polynomial as a function of diffraction angle. A polynomial function fitting was then applied to the FWHM results to obtain the best experimental behaviour. Fig. 8 ▸ shows the Si diffraction pattern for the NIST Si 640f standard measured at 16 keV. A negligible asymmetrical contribution was observed over the range of diffraction angles; this is visualized in Fig. 9 ▸.

**Figure 10:**
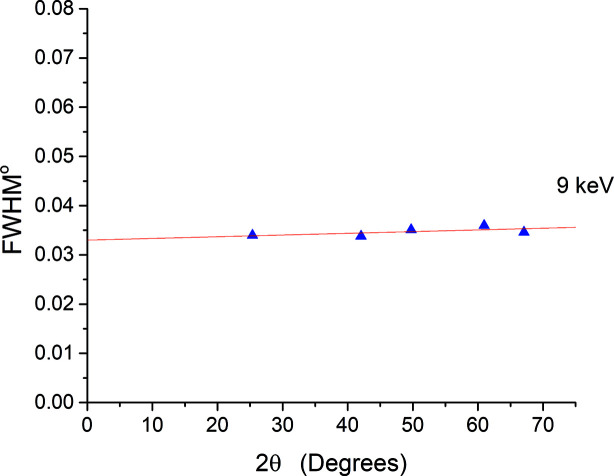
Instrumental angular resolution function measured by Si (NIST 640f) at 8 keV.

**Figure 11:**
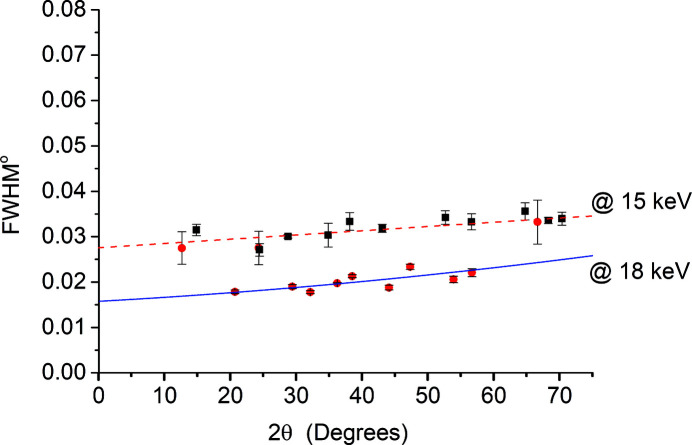
Instrumental angular resolution function measured by Si (NIST 640f) at 15 and 18 keV.

At 8 and 15 keV, the FWHM was observed to behave linearly with 2theta varying by about 0.03° over the full range (Fig. 10 ▸). This value decreases to 0.015° over the full range of 2theta when the energy increases to 18 keV (Fig. 11 ▸). Therefore higher energies can be used for those experiments that require high angular resolution. Although further improvement in the angular resolution is obtained if the crystal analyzer setup is used (Gozzo *et al.*, 2004 ▸), the obtained results present a reasonably good compromise taking advantage of the fast experimental time that the detector provides.

## 4. Case study: line profile analysis for nanocrystalline fluorites

One of the main applications of XRPD is line profile analysis (LPA) for investigating the microstructure of materials and their relationships with the properties of materials. To apply LPA, it is necessary to obtain the best instrumental profiles in terms of shape and FWHM. Ball-milled CaF2 is taken as a case for study to use MS for investigating the effect of ball milling on the microstructure of CaF2. Pristine CaF2 powder was milled for 30 h using a planetary mill machine (Fritsch Pulverisette 6). The milled powder was then filled in a 0.5 mm borosilicate capillary and measured at 18 keV with a 1 s acquisition time at each detector frame. 5° from each frame and the overlaps between frames were removed in the data extraction process, with one data file for each frame, while these data files were then merged to give one file ready for further analysis.

A Whole Powder Pattern Modelling (WPPM) approach (Scardi & Leoni, 2002 ▸, 2004 ▸) was used to analyse the milled powder, assuming the crystallites of the powder to be distributed log-normally with spherical shapes, as the most expected shapes produced from ball milling. The log-normal distribution is defined by two refinable parameters, *i.e.* mean (μ) and variance (σ). Beside the size model, the anisotropic lattice strain model used to define the dislocation created by ball milling includes mean dislocation density (ρ), effective outer cut-off radius (*R*_e_) and mixing parameter (fraction of edge/screw dislocations, *f*E). Together with the size and strain model, the addition of the lattice parameters of the CaF2 face-centred cubic unit cell, together with peaks reflections *hkl*, was necessary. A Chebyshev function was also added in the model to fit the background of the diffraction pattern. Lattice parameters, size distribution parameters (μ and σ), strain model parameters, background coefficients and peak intensities were considered as refinable parameters all together simultaneously using the least-squares fitting approach though the *PM2K* software (Leoni *et al.*, 2006 ▸).

**Figure 12:**
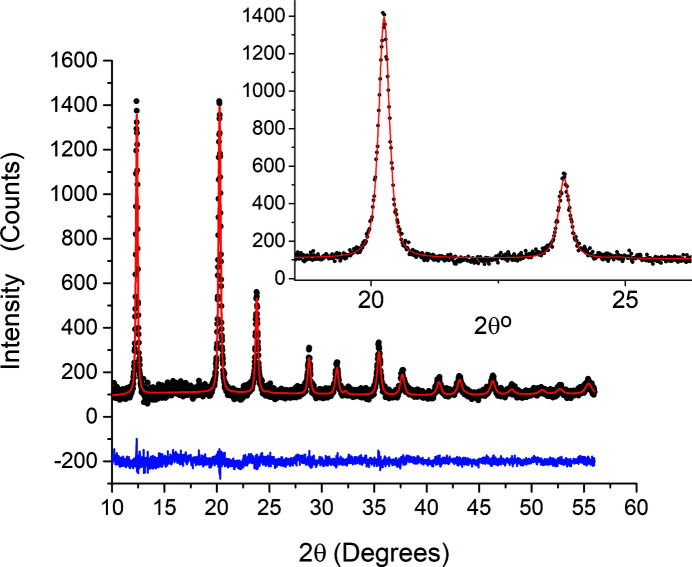
Whole Powder Pattern Modelling (WPPM) fitting for ball-milled CaF2. Experimental data are circles and the refined model is a line. The difference plot is shown below.

**Figure 13:**
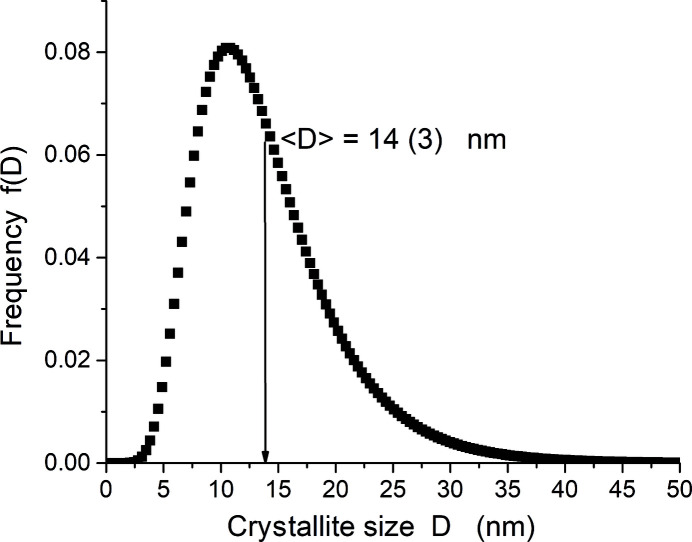
Crystal size distribution for ball-milled CaF2.

Table 3 ▸ reports the main results of the best refined model. Fig. 12 ▸ shows the WPPM result for the CaF2 sample revealing the goodness-of-fit and a good match between the refined model and the experimental data, while Fig. 13 ▸ shows the refined log-normal crystallite size distribution.

## 5. Conclusion

In this article, a full description for the SESAME MS beam­line for X-ray powder diffraction applications has been reported. The layout of the main components of the front-end and optics were highlighted and, in particular, the instrumental parameters, in terms of beam size at the experimental stations. A set of Si standard measurements were performed to obtain the instrumental resolution as a function of the diffraction angle, from which a small contribution was found, especially with increasing energy. The diffraction experiment at the MS beam­line was collected using the PILATUS 300K detector donated by DECTRIS based on solid-state Si microstrips arrays which provide a good compromise between high angular resolution with fast read-out time which is especially efficient for time-resolved experiments. A case study was presented for the line profile analysis of ball-milled fluorites to obtain microstructure details in terms of crystallite size distribution and lattice defects density.

## Figures and Tables

**Table 1:** The major MS beam­line parameters

Energy range (keV)	5–25
Accepted divergence (mrad)	0.23 (V) × 1.5 (H)
Flux at the sample at 10 keV (photons s^−1^)	10^13^
Energy resolution (eV)	2
Effective beam size at the sample (FWHM) (µm)	300 (V) × 2800 (H)

**Table 2:** The main components of the beam­line and their corresponding distances from the wiggler centre

Component	Distance (m)
Wiggler centre	0
Fixed mask	8.1
Photon shutter	8.4
Rotating filter (glassy carbon)	9.7
White beam slits	10.2
Bremsstrahlung stopper	11.4
Be windows	13.57
Fast absorber	14.1
Filters	14.3
Collimating mirror	15.5
Wire beam position monitor (BPM)	16.6
Double Si crystal monochromator	17.5
Focusing mirror	19.9
Monochromatic slits	21.8
Last slits	33.0
Diffractometer (sample location)	33.5

**Table 3:** The main results of the best refined model

Parameter	Refined values
Unit-cell parameter (nm)	0.54663 (5)
〈*D*〉 (nm)	14 (3)
ρ (× 10^15^ m^−2^)	9 (2)
*R*_e_ (nm)	6 (1)
mixp	0.1 (1)
Goodness-of-fit	0.94
